# In vitro study of TSC1 deficiency in preadipocytes: insights into development and treatment options for tuberous sclerosis related lipomatosis

**DOI:** 10.1186/s13023-026-04442-y

**Published:** 2026-06-12

**Authors:** Julika E. Friedrich, Julia Hentschel, Sandy Richter, Henriette Kiep, Maria Arélin, Konrad Platzer, Torsten Schulz, Andreas Merkenschlager, Wieland Kiess, Steffi Mayer, Rami Abou Jamra, Diana Le Duc, Antje Garten, Anna S. Kirstein

**Affiliations:** 1 Center for Pediatric Research, University Hospital for Children & Adolescents, Leipzig University, Leipzig, Germany; 2 Institute of Human Genetics, Leipzig University, Leipzig, Germany; 3 University Hospital for Children and Adolescents, Leipzig, Germany; 4 Department of Orthopedic, Trauma and Plastic Surgery, Leipzig University Hospital, Leipzig, Germany; 5 University Hospital for Pediatric Surgery, Leipzig, Germany; 6 German Center for Child and Adolescent Health (DZKJ), partner site Leipzig/Dresden, Leipzig, Germany

**Keywords:** Tuberous sclerosis, Lipoma, TSC1, mTOR, Sirolimus, Alpelisib

## Abstract

**Background:**

Tuberous sclerosis complex (TSC) is a rare genetic neurocutaneous disorder resulting from mutations in the *TSC1* or *TSC2* genes, characterized by overgrowth and lesions in multiple organs. While renal angiomyolipomas are commonly seen, lipomas located elsewhere are rarely reported in these patients.

**Results:**

We identified a heterozygous *TSC1* mutation in a pediatric patient, who developed a lipoma in the gluteal region, which recurred after surgical resection. We observed a loss of heterozygosity in the lipoma tissue, resulting in TSC1 deficiency and subsequent activation of the mechanistic target of rapamycin (mTOR) signaling pathway. Further in vitro experiments showed that silencing *TSC1* in adipocyte progenitors led to increased cell proliferation, supporting the hypothesis that *TSC1* deficiency contributes to lipoma formation. Treatment with mTOR inhibitors, such as sirolimus and torin-1, as well as the phosphoinositide 3-kinase (PI3K) inhibitor alpelisib reduced cell proliferation and pathway activation in *TSC1*-deficient cells.

**Conclusions:**

This study highlights the need for further investigation into the efficacy of pathway inhibitors in managing TSC-related lipomas in vivo and offers a potential treatment avenue for patients suffering from recurrent lipomatosis.

**Supplementary Information:**

The online version contains supplementary material available at 10.1186/s13023-026-04442-y.

## Background

As a genetic neurocutaneous multisystem disorder, tuberous sclerosis complex disorder (tuberous sclerosis) is characterized by the development of overgrowth and lesions in various organs, including the skin, brain, kidneys, heart and lungs [1]. The incidence rate is estimated between 1:6,000 and 1:10,000 live births, making it a relatively rare condition [1]. Tuberous sclerosis is caused by a deficiency in either hamartin/tuberous sclerosis complex (TSC) 1 or tuberin/TSC2. While *de novo* mutations are responsible for two-thirds of the diagnoses, the remaining one-third underlies an autosomal dominant inheritance [2].

While renal angiomyolipomas occur often, lipomas, which are benign tumors composed of adipose tissue, are rarely described in patients with tuberous sclerosis.

Here we report on a pediatric patient with tuberous sclerosis caused by a heterozygous variant in *TSC1* who developed a lipoma in the right gluteal region. In patients with tuberous sclerosis, only seven cases of diffuse lipomatosis are described with lipomas mostly located in the lower limbs. Although some of these patients underwent surgical resection, such as lipectomy or liposuction, a high rate of postoperative local recurrence has been reported [3–7]. Genetic testing of the adipose tissue samples was not performed in the reported cases. The underlying mechanisms of lipoma formation need further investigation.

**Fig. 1:**
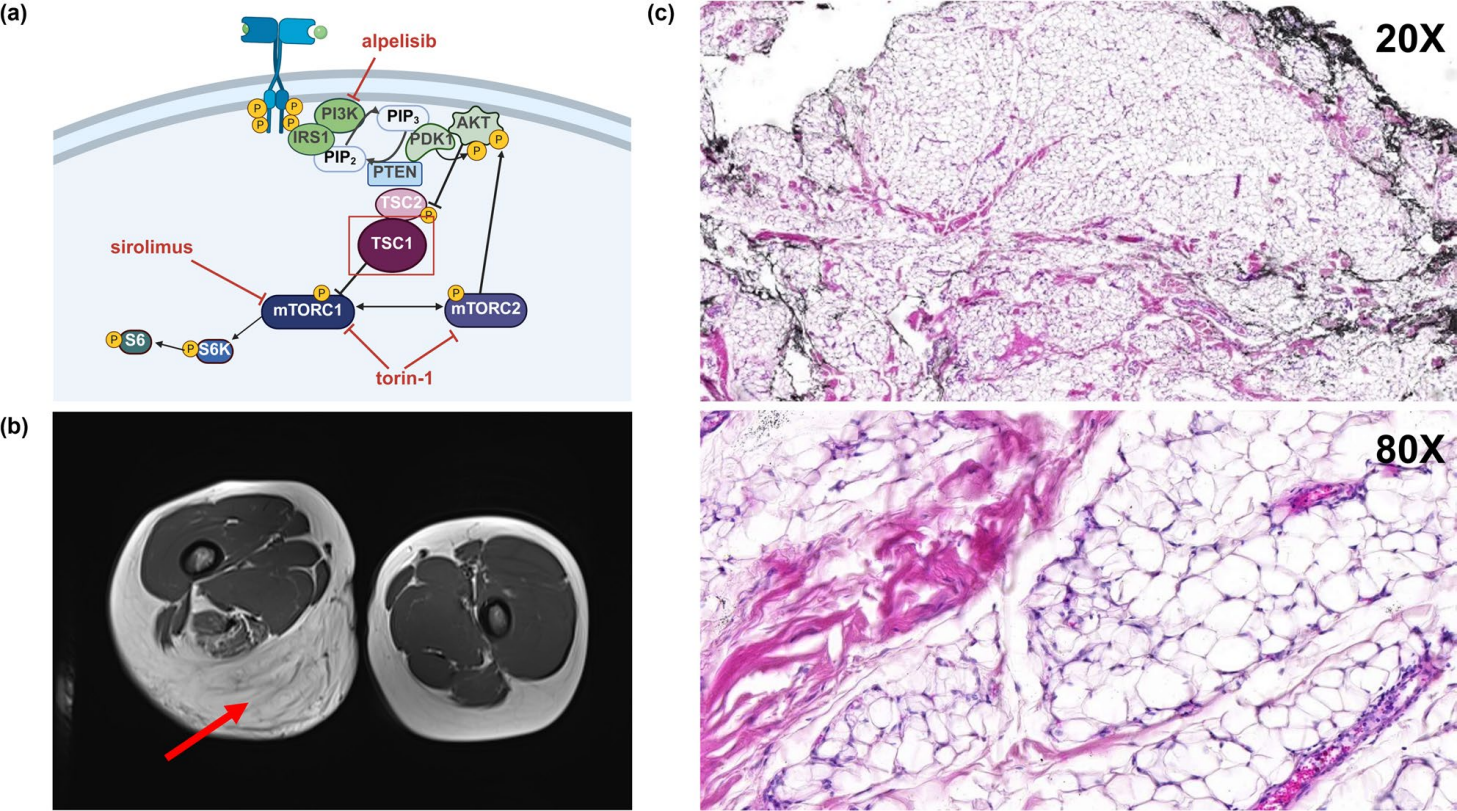
Presentation of the patient. **(a)** Simplified mTOR signaling pathway with inhibitors and targets. The TSC complex inhibits mTOR, leading to increased mTOR activation in absence of TSC1. While alpelisib inhibits the PI3K upstream mTOR, sirolimus targets mTORC1, and torin-1 inhibits mTORC1 and mTORC2 **(b)** An MRI showed subcutaneous and intramuscular lipomatous tissue subcutaneous in the right gluteal region (red arrow) **(c)** Microscopic examination demonstrated an angiolipoma. The images were taken at 20x and 80x magnification

TSC1 and TSC2 form the heterodimeric TSC protein complex that inhibits signal transduction to mechanistic target of rapamycin complex 1 (mTORC1) acting as a tumor suppressor complex. Thus, the deficiency in either TSC1 or TSC2 leads to a constitutive mTOR pathway activation. The signaling molecule AKT is activated by numerous growth factors through the insulin receptor substrate 1 (IRS-1) and phosphoinositide-3-kinase (PI3K) and suppresses TSC via phosphorylation. Once TSC is inhibited, mTOR complex 1 (mTORC1) is activated via Ras homologue enriched in brain (Rheb) followed by the phosphorylation of multiple targets such as ribosomal protein S6 Kinase B1 (p70S6K/S6K) and subsequently ribosomal protein S6 (S6) (Fig. 1a).

In patients with phosphatase and tensin homolog (PTEN) hamartoma tumor syndrome (PHTS), hyperactivation of mTOR as a major regulator of cell growth and proliferation contributes to the development of lipomas [8]. Treatment with inhibitors, such as mTOR inhibitor sirolimus (rapamycin) and PI3K inhibitor alpelisib can reduce growth of lipoma cells, highlighting the therapeutic potential of targeting the mTOR signaling pathway [9].

We investigate mechanisms leading to aberrant adipose tissue growth in relation to TSC1 downregulation in vitro and test whether different mTOR pathway inhibitors could reverse abnormal growth of TSC cell models to evaluate their potential for treatment of tuberous sclerosis-associated lipomas.

## Materials and methods

### Genetic analysis

Strictly following manufacturer’s instructions and SOPs, library preparation was done using the Nextera DNA Flex Pre-Enrichment LibraryPrep with lllumina Nextera DNA UD Indexes by lllumina. Target enrichment was achieved by using the Human Core Exome hybridization probes from Twist Bioscience. Paired-end Next-Generation-Sequencing was then performed on a NovaSeq 6000 Instrument using an S l Reagent Kit (300 cycles) by lllumina.

Analysis of the raw data was performed using the software Varfeed (Limbus Medical Technologies GmbH, Rostock) and the variants were annotated and prioritized using the software Varvis (Limbus). We have prioritized all potential protein-influencing variants with regard to their pathogenicity and clinical relevance according to all possible inheritance modes.

We performed a genome wide karyotyping of copy number variations (CNV’s) with the tool Varfeed^®^ CNV (Limbus). Components of the algorithm were based on the publication of Talevic et al. [10]. Clinical relevant CNV’s were validated with a second method (e.g. quantitative PCR (qPCR), multiplex ligation-dependent probe amplification). Nomenclature of reported CNV’s is according to ISCN 2020.

### MR and microscope images

A T1-weighted MR image was obtained using the turbo spin echo technique in the transverse plane. The microscopy images were obtained after embedding the resected lipoma in paraffin, sectioning and staining with hematoxylin and eosin.

### Cell culture

Human preadipocytes were cultured in Dulbecco’s modified Eagle’s medium (DMEM)/F12 medium supplemented with 10% fetal calf serum, glutamine (2 mM), D-biotin (33 mM) and D-pantothenic acid (17 mM) at 37 °C in a humidified atmosphere containing 5% CO_2_. SGBS cells are a human preadipocyte cell strain derived from the stromal fraction of subcutaneous adipose tissue obtained from an infant with Simpson-Golabi-Behmel syndrome [11]. We used SGBS cells starting from generation 39, which corresponds to 78 days in culture. Patient’s stromal vascular fraction (SVF) cells were isolated from a resected piece of lipoma tissue. Control SVF cells were isolated from visceral adipose tissue resected during bariatric surgery of obese, non-diabetic adult donors. Isolation and culture was performed as previously described [8]. Detailed information about the control SVF cells is listed in Additional file 1, Table S1.

### Small-interfering RNA (siRNA) mediated knockdown of TSC1

SGBS cells were seeded one day before transfection at a density of 1,400 cells/cm^2^. The transfection was performed using the NEON transfection system (Invitrogen, Thermo Fisher Scientific, Inc) as previously described [8]. Cells were harvested *via* trypsinization and washed with DPBS. A combination of three TSC1 siRNAs (ID #s14433, #s14434, #s14435, all Ambion, Thermo Fisher Scientific, Inc) or control siRNA (Silencer™ Negative Control No. 1 siRNA, Ambion) was used at a final concentration of 10 nM in the culture medium after transfection. Pellets were resuspended in 100 µl R-buffer for transfection and electroporated in Neon 100 µl tips at 1300 V, 2 pulses, 20 ms. Transfected cells were distributed to multi-well tissue culture plates for functional assays. Knockdown efficiency was determined via Western blot.

### Proliferation assay

After transfection, cells were seeded at a density of 1,500 cells/well on 96-well plates in culture medium. The medium was replaced at day 1 (one day after transfection) and day 4 using either solvent control (DMSO) or different inhibitors in culture medium. The following inhibitors were used: mTORC1 inhibitor sirolimus (100 nM), torin-1 (100 nM) as inhibitor of both mTORC1 and mTORC2, and alpelisib (10 µM) as PI3K inhibitor. 100 nM Sirolimus and 10 µM alpelisib were used alone or in combination. At day 1, day 4 and day 8 after transfection cells were fixed in 4% paraformaldehyde. Nuclei were stained using Hoechst 33,342 for 5 min at a concentration of 1 µg/mL in DPBS. Images were taken with the Evos FL Auto 2 Cell Imaging System (Thermo) at 100x magnification (automatic scan of 50% of the well area in DAPI channel, 6 wells per condition). Nuclei were counted automatically using ImageJ/Fiji [12].

### Cell viability

Cells were treated with inhibitors for 72 h as described above and cell viability was estimated using the WST-1 assay (Roche, Mannheim, Germany) following the manufacturer’s instruction. Absorbance was measured at 450 nm 1 h after adding WST-1 reagent.

### Adipocyte differentiation

Differentiation assays and lipid staining were performed as previously described [8]. The medium was changed to differentiation medium one day after transfection (DMEM/F12 containing 8 mg/mL D-biotin, 10 mg/mL D-pantothenic acid, 2 µM rosiglitazone, 25 nM dexamethasone, 0.5 mM methylisobutylxanthine, 0.1 µM cortisol, 0.01 mg/mL apotransferrin, 0.2 nM triiodotyronin and 20 nM human insulin). After 8 days of adipose differentiation, cells were fixed, stained with Nile red lipid stain (0.5 g/mL, Sigma) and Hoechst 33,342. Fluorescence microscope pictures were taken using the Evos FL Auto 2 Cell Imaging System (Thermo), and cell counting was performed with the Celleste Image Analysis Software (Thermo). RNA isolation from differentiated SGBS control and TSC1 knockdown SGBS cells was performed using RNeasy Mini Kit (Qiagen, Hilden, Germany). Reverse transcription and qPCRs were performed as previously described [13]. Additional file 1, Table S2 contains the list of primers used for qPCR assays. Results were normalized to housekeeping genes hypoxanthine phosphoribosyltransferase (*HPRT*) and TATA box–binding protein (*TBP*).

### Western blot analysis

For Western blot analyses, cells were seeded at a density of 150,000 cells/well on 6-well plates. To asses pathway activation in control compared to TSC1 KD cells, the medium was replaced with serum free medium 24 h post transfection and harvested for protein extraction on the next day. To test the effect of inhibitors cells were incubated with medium containing the solvent control (DMSO) or various inhibitors. After lysis, electrophoretic separation of proteins, and blotting onto nitrocellulose membranes, immunoblotting was performed using standard protocols. For Western blots, 15–20 µg protein per lane was used. The membranes were incubated with primary and secondary antibodies as listed in Additional file 1, Table S3. We used α-tubulin (tubulin) as loading controls. For probing of total proteins and housekeepers, membranes previously stained for phosphorylated proteins were stripped using a buffer containing 2% SDS, 62.5 mM Tris-HCl (pH 6.8), and 0.8% β-mercaptoethanol. Membranes were incubated 30 min shaking at 50 °C in the stripping buffer for complete removal of primary antibodies, subsequently washed, blocked, and reprobed. Densitometric analyses were performed using Image [8].

### Immunofluorescence staining

Ki-67 immunofluorescence staining and phosphorylated S6 (pS6) immunofluorescence staining were performed using the antibodies listed in Additional file 1, Table S3. 4 days after transfection, cells were fixed in 4% paraformaldehyde, permeabilized and blocked in IF-buffer (PBS + 5% BSA + 0.3% Tween20) for 1 h at room temperature. Cells were stained with the primary antibody overnight at 4 °C. Cells were washed three times with IF-buffer and incubated with a secondary antibody for 2 h at room temperature in the dark. After washing (2x IF buffer, 1x PBS), nuclei were stained with Hoechst 33,342. Inhibitors were used for 3 days in the same concentration as listed above. The proliferative fraction was defined as Ki-67 positive cells per Hoechst nuclei count in %.

### Statistical analysis

Means of ≥ 3 independent experiments were statistically analyzed using GraphPad Prism 10 (GraphPad Software Inc.). For comparison of two conditions, means of independent experiments were compared via paired Student’s t-test. For multiple comparisons, we used one-way analysis of variance (ANOVA) followed by a Dunnett’s multiple comparisons test or by Šidák’s multiple comparisons test. Results are presented as mean ± SEM.

## Results

The data underlying the figures presented in this manuscript can be found in Additional file 2, Table S4.

### Patient’s lipoma shows a loss of TSC1

The patient presented with a tumor located in the right gluteal region. MR imaging revealed the presence of subcutaneous and intramuscular lipomatous tissue in the right gluteal area, extending into the gluteal, semitendinosus, and biceps femoris muscles, (Fig. 1b), microscopic examination of resected tissue demonstrated an angiolipoma (Fig. 1c). To date, the patient underwent two liposuction procedures (in August 2023 and March 2024) due to the recurrence of the lipoma.

Furthermore, the patient exhibited additional manifestations including a developmental disorder, subependymal nodules and multiple tubers within the brain, along with cutaneous manifestations such as white spots and shagreen patches. We identified a likely pathogenic variant [14] in the *TSC1* gene (9:135796747; NM_000368.5:c.737 + 3 A > G, r.664 _737del, p.(Pro222Valfs*8)). A segregation analysis was conducted within the family, showing a paternal inheritance of the variant. The father is asymptomatic. The patient has one affected sister carrying the same variant, showing mild CNS involvement, and hemangiomas on hands, back, and head. Lipomas were not reported in any other family member.

mRNA analysis confirmed the presence of aberrant splicing. In accordance with tuberous sclerosis genetic testing recommendations, we also sequenced the intronic regions, which revealed no pathogenic variants.

**Fig. 2:**
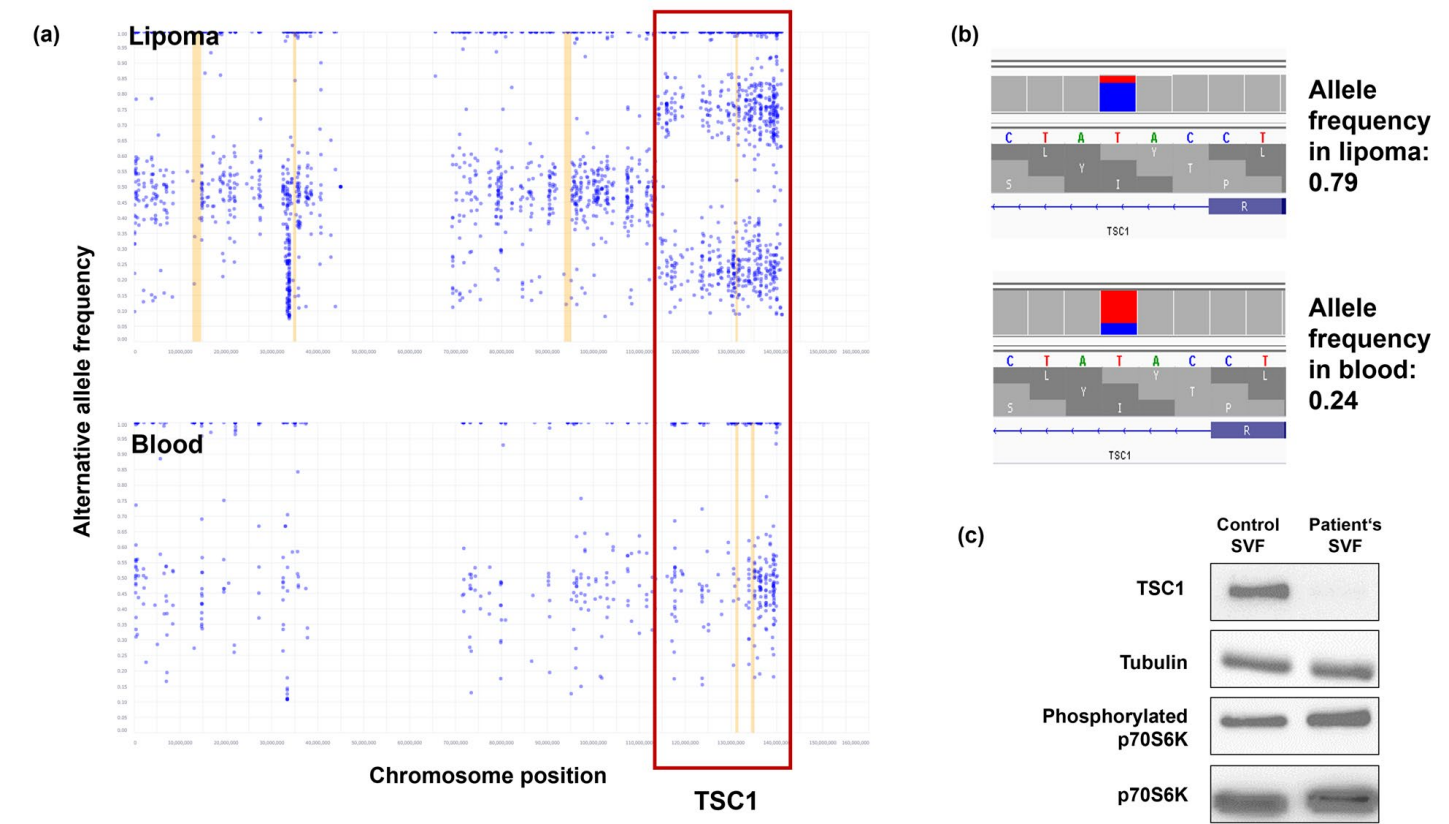
TSC1 in patient’s lipoma. **(a)** Allele frequency distributions on chromosome 9 in lipoma and blood. Blood analysis was performed using the TruSight One Panel (Illumina, ~ 4800 genes), while lipoma analysis utilized the Twist Exome v2. The alternative allele frequencies for all variants on chromosome 9 are plotted. In the lipoma sample, around position 110,000,000 on chromosome 9, a polarization of alternative allele frequencies (< 0.35 and > 0.70) is observed, with a loss of variants with a frequency around 0.5. This suggests allele deletion and loss of heterozygosity. Red box highlights the region of loss of heterozygosity in lipoma, encompassing *TSC1. ***(b)** Alternative allele frequency of the variant in *TSC1* in lipoma (allele frequency of 0.79) and blood (allele frequency of 0.24). **(c)** Representative Western blot image (*n* = 2) of patient’s SVF cells compared to SVF cells of healthy donors (control): Patients lipoma cells show a loss of TSC1 protein resulting in increased phosphorylation of the mTOR signaling pathway target p70S6K. Original blots are presented in Additional file 1, Fig. S1

In conjunction with the clinical findings, the diagnosis of tuberous sclerosis could be established. While allele frequency analysis showed a heterozygous variant in blood, we observed a loss of heterozygosity within the lipoma (Fig. 2a and b).

To further investigate whether the patient’s lipoma might be related to a TSC1 loss of function, we performed Western blot analysis of patient’s SVF cells (Fig. 2c, Additional File 1, Fig. S1) isolated from the lipoma. We detected a loss of TSC1 protein in comparison to cells extracted from adipose tissue of healthy donors. In addition, we observed an increased phosphorylation of mTOR target p70S6K compared to control cells, suggesting that the mTOR signaling pathway is overactivated in the patient’s lipoma.

### TSC1 knockdown increases proliferation and mTOR pathway activation in SGBS cells

**Fig. 3:**
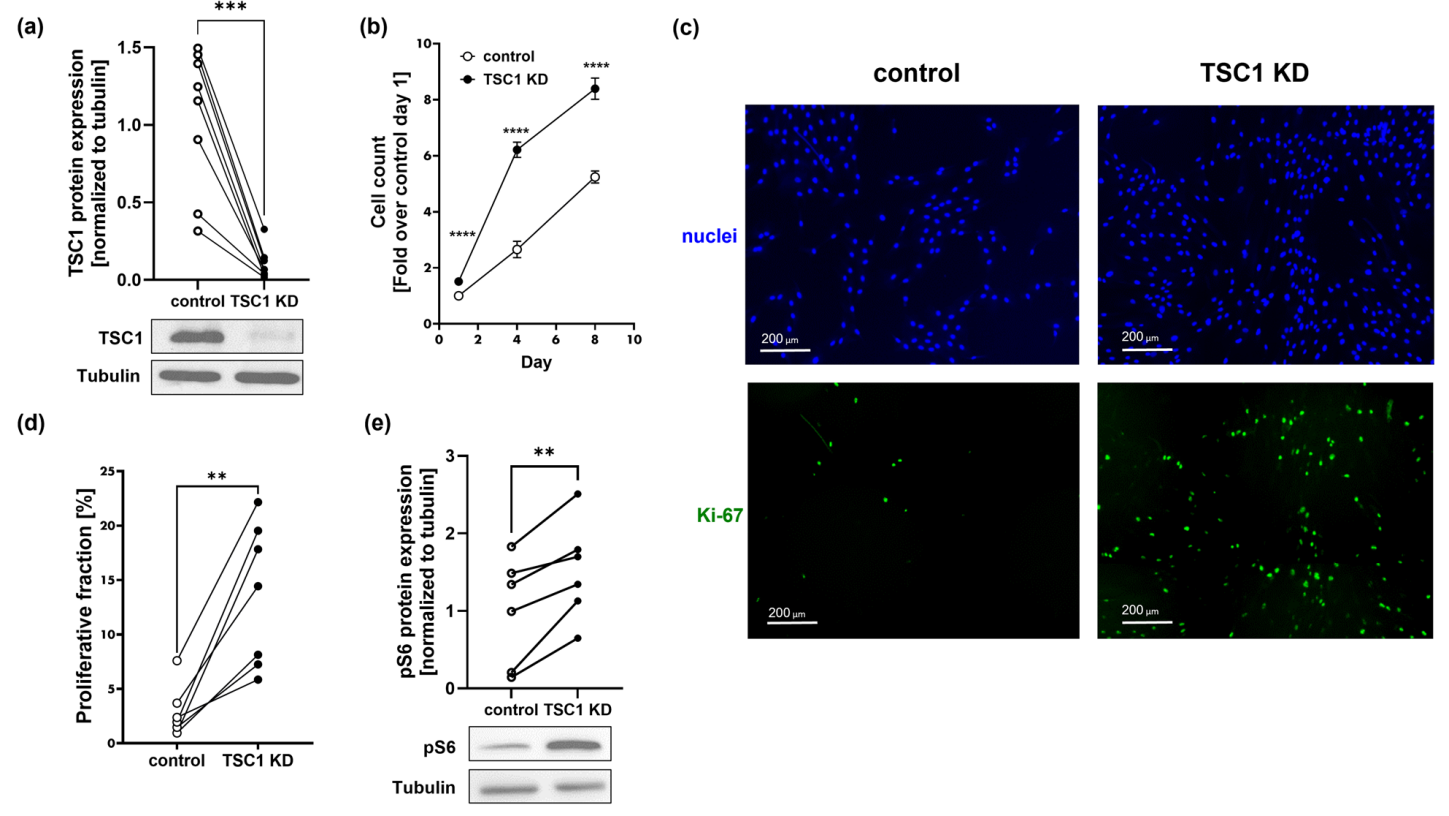
TSC1 downregulation enhances proliferation and mTOR signaling in SGBS cells. (a) Western blots of control and TSC1 KD SGBS cells: TSC1 protein expression was reduced in TSC1 KD cells. (normalized to α-Tubulin, n = 8, ***p < 0.001) showing the successful KD via siRNA. Original blots are presented in Additional file 1, Fig. S2 and Additional file 1, Fig. S3. **(b)** Hoechst nuclei staining of control and TSC1 KD cells: Cell count in TSC1 KD cells was increased on day 1, 4 and 8 after transfection. (*n* = 10, ****p < 0.0001, mean ± SEM, p-values were determined using raw data). Microscopy images at 100x magnification **(c)** and quantification **(d)** of Ki-67 immunofluorescence staining in control and TSC1 KD cells 4 days after transfection: TSC1 KD cells showed a 5.01-fold increase of proliferation marker Ki-67 positive cells compared to control (*n* = 7, **p < 0.01). (e) Western blots of control and TSC1 KD SGBS cells after 24 h serum free incubation: Phosphorylated (p) S6 protein expression was higher in TSC1 KD cells compared to controls (normalized to α-Tubulin, n = 6, **p < 0.01) showing the activation of the mTOR signaling pathway. Western blot panels show one representative image and densitometric analyses. p-values were determined via paired t-test. Original blots are presented in Additional file 1, Figure S4 and Additional file 1, Fig. S5

To investigate a potential role of TSC1 in lipoma formation, we performed a knockdown of TSC1 in the human preadipocyte cell model SGBS [11] and examined the impact on proliferation, adipocyte differentiation and mTOR signaling pathway activation. A nearly complete TSC1 knockdown was confirmed at the protein level via Western blot analysis (*p* = 0.0003; Fig. 3a, Additional File 1, Fig. S2-S3).

Proliferation assays revealed an increase of proliferation in TSC1 knockdown cells compared to controls. On day 1 after transfection the cell count in knockdown cells was 1.51 ± 0.04 fold increased compared to control cells (*p* < 0.0001; Fig. 3b). On day 4 the cell count was 6.210 ± 0.180 fold increased in TSC1 knockdown cells compared to a 2.65 ± 0.29 fold increased cell count in control cells. On day 8 we observed an 8.39 ± 0.25-fold increase in cell count of TSC1 knockdown cells compared to a 5.24 ± 0.21 fold increase of the controls (*p* < 0.0001; Fig. 3b).

Ki-67 immunofluorescence staining showed a 5.01 ± 0.91 fold increase of the proliferative fraction in TSC1 knockdown cells on day 4 compared to control (*p* = 0.0022; Fig. 3c-d).

After 24 h serum free incubation Western blot analysis revealed a 1.52 ± 0.26 fold higher phosphorylated downstream mTOR target ribosomal protein S6 in TSC1 knockdown cells compared to control (*p* = 0.0034; Fig. 3e, Additional File 1, Fig. S4-S5), indicating an mTOR pathway activation.

We did not observe pronounced effects on the adipogenic differentiation of SGBS cells. Nile red lipid staining showed no differences in the number of differentiated cells (Additional file 1, Fig. S6a-b). Furthermore, we did not observe a difference between differentiated control and TSC1 knockdown cells in mRNA expression of adipocyte differentiation markers adiponectin, peroxisome proliferator-activated receptor gamma (*PPARG*) and fatty acid synthase **(***FASN*) (Additional file 1, Fig. S1c). Western blots of PPARγ showed a subtle induction of the differentiation marker at the protein level (*p* = 0.002) while increase of FASN (*p* = 0.07) and adiponectin (*p* = 0.83) was not significant (Additional file 1, Fig. S6d). Knockdown efficiency remained stable after 8 days of differentiation as shown via Western blot (Additional file 1, Fig. S6d).

### Treatment with mTOR pathway inhibitors reduced proliferation and pathway activation

To assess whether inhibition of the mTOR pathway might be beneficial for patients with a tuberous sclerosis-related lipoma, we tested the effects of sirolimus, alpelisib and torin-1 in vitro on TSC1 knockdown SGBS cells. Proliferation assays, Ki-67 staining and cell viability assays revealed a decrease in proliferation with inhibitor treatment, suggesting that a treatment with pathway inhibitors might affect lipoma growth.

**Fig. 4:**
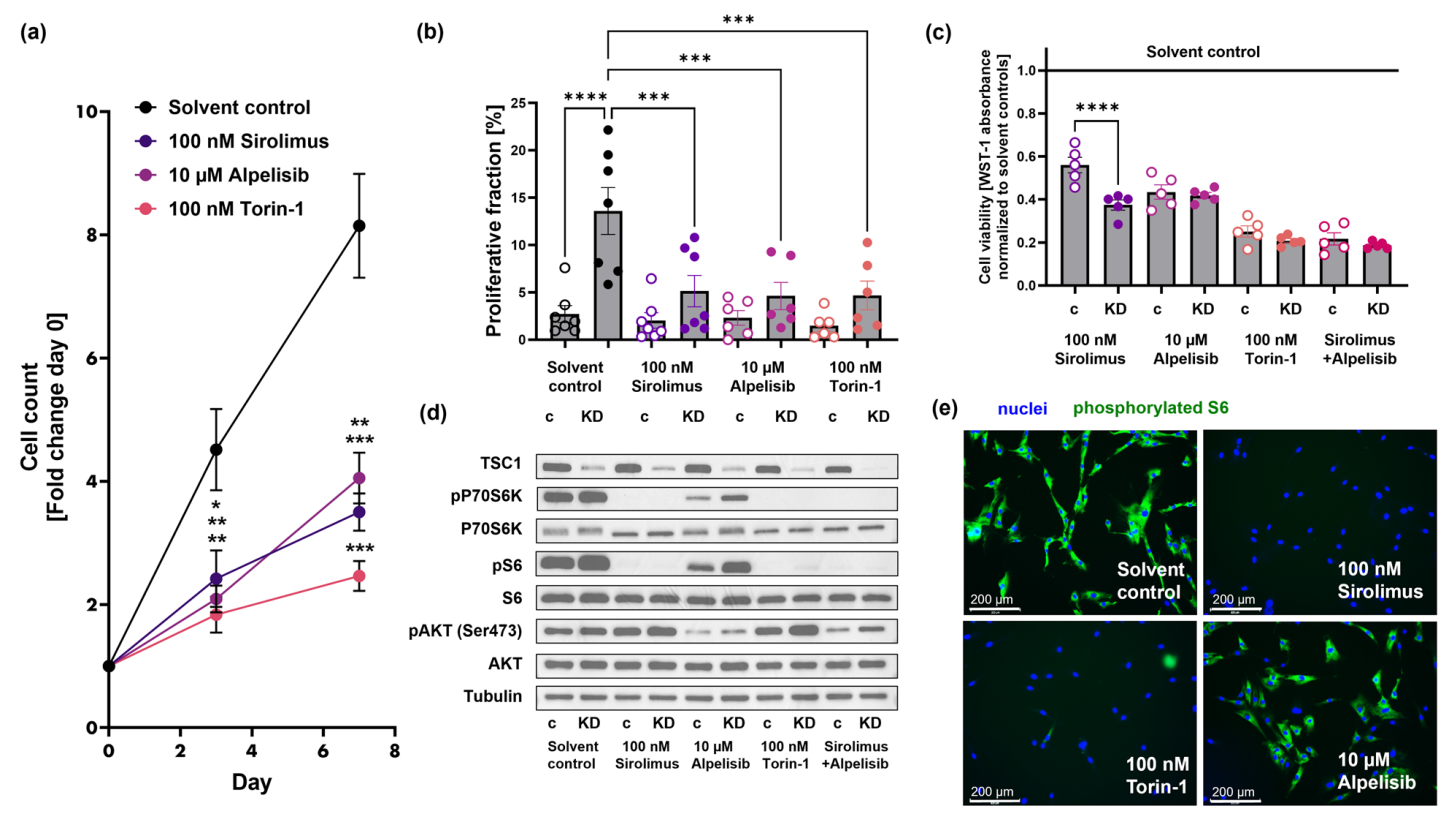
Inhibitor treatment of TSC1 KD SGBS cells reduces proliferation, cell viability and mTOR pathway activation. **(a)** Hoechst nuclei staining of inhibitor treated TSC1 KD cells: Cell count in treated cells was decreased after 3 and 7 days of inhibitor treatment compared to solvent control (*n* = 4 on day 3, *n* = 3 on day 7, *p < 0.05, **p < 0.01, ***p < 0.001, mean ± SEM). (**b**) Ki-67 immunofluorescence staining after 48 h of inhibitor treatment: Cells treated with inhibitors showed a decrease of the proliferative fraction compared to solvent controls (***p < 0.001, ****p < 0.0001, mean ± SEM). We observed no significant differences between solvent control and inhibitor treatments in control cells . (**c**) WST-1 cell viability assays after 72 h of inhibitor treatment: Compared to solvent controls (black line), viability decreased in cells treated with sirolimus, alpelisib, torin-1, or combinations of 100 nM sirolimus and 10 nM alpelisib, both for control and TSC1 KD cells (p < 0.001 for all treated groups compared to the respective solvent controls). We observed a significant decrease in cell viability for TSC1 KD cells compared to controls treated with sirolimus (n = 5, ****p < 0.0001, mean ± SEM). **(d)** Representative Western blot of SGBS control (c) and TSC1 KD (KD) cells: Inhibitor treatment led to specific pathway downregulation as shown via phosphorylated (p) p70S6K, S6 and AKT Western blots. While sirolimus and torin-1 were potent in inhibiting p70S6K and S6 phosphorylation, alpelisib inhibited AKT phosphorylation, which was induced upon sirolimus treatment. A combination of sirolimus and alpelisib inhibited phosphorylation of p70S6K, S6 and AKT. Original blots and densitometry are presented in Additional file 1, Fig. S8-11 (*n* = 3). **(e)** Hoechst nuclei staining (blue) and phosphorylated S6 immunofluorescence staining (green): TSC1 KD cells treated with inhibitors show lower activation of S6 compared to solvent controls. Alpelisib was less effective in reducing S6 activation compared to sirolimus and torin-1. p-values were determined via one-way ANOVA followed by Dunnett’s multiple comparisons test or by Šídák’s multiple comparisons test

After 3 days of inhibitor treatment, proliferation assays of TSC1 knockdown cells showed a significant decrease in cell count with torin-1 (*p* = 0.0027), sirolimus (*p* = 0.0146) and alpelisib (*p* = 0.0056) (Fig. 4a). Cell count in treated cells was decreased from 8.15 ± 0.84 fold higher in solvent control to 3.50 ± 0.30 fold with sirolimus (*p* = 0.0005), 4.05 ± 0.41 fold with alpelisib (*p* = 0.0012) and 2.46 ± 0.24 fold with torin-1 (*p* = 0.001) 7 days after inhibitor treatment, all compared to solvent control day 1. The tested inhibitors also decreased cell count in control cells without TSC1 knockdown as shown in additional file 1, Fig. S7, but the effect was less pronounced. Proliferation in treated cells without TSC1 knockdown was decreased from 3.87 ± 0.40 fold in solvent control to 2.55 ± 0.34 fold with sirolimus (*p* = 0.03), 3.03 ± 0.17 fold with alpelisib (not significant) and 2.05 ± 0.29 fold with torin-1 (*p* = 0.01) 7 days after inhibitor treatment, all compared to solvent control day 1.

After 3 days of inhibitor treatment, the proliferative fraction (Fig. 4b) was reduced from 13.60 ± 2.47% in solvent controls to 5.14 ± 1.65% in cells treated with sirolimus (*p* = 0.0006), 4.62 ± 1.44% in cells treated with alpelisib (*p* = 0.0005) and 4.68 ± 1.52% in cells treated with torin-1 (*p* = 0.0005). We observed no significant differences between solvent control and inhibitor treatments in control cells without TSC1 knockdown (Fig. 4b).

Cell viability/metabolic activity was measured via WST-1 assay (Fig. 4c) of SGBS TSC1 knockdown cells after 3 days of inhibitor treatment and was reduced by 63 ± 2% in cells treated with sirolimus, by 58 ± 1% in cells treated with alpelisib, by 79 ± 1% in cells treated with torin-1, and by 81 ± 1% in cells treated with a combination of sirolimus and alpelisib compared to solvent controls (black line, *p* < 0.0001 for all conditions). Of note, all inhibitors also significantly reduced cell viability/metabolic activity in controls without TSC1 knockdown compared to solvent controls (*p* < 0.0001 for all conditions). While the reduction of cell viability was less pronounced for sirolimus in cells without TSC1 knockdown (*p* < 0.0001 for difference between control and TSC1 knockdown), there was no significant difference between controls and TSC1 knockdown cells in all other conditions.

Western blot analysis of TSC1 knockdown cells (Fig. 4d, Additional file 1, Fig. S8-S11) revealed decreased phosphorylation of the downstream mTOR targets S6 and p70S6K after a three-day treatment with sirolimus, torin-1, alpelisib or a combination of sirolimus and alpelisib (*p* < 0.0001 for all conditions compared to solvent controls). This suggests that the observed upregulation of the mTOR pathway was attenuated by the inhibitors. Both mTOR inhibitors, sirolimus and torin-1, were more effective in inhibiting the phosphorylation of mTOR downstream targets compared to alpelisib, which had a lesser effect on attenuating activation of mTOR downstream targets. The increase in P70S6K phosphorylation in TSC1 knockdown cells was still detectable in alpelisib treated cells (*p* = 0.04), while phosphorylation was completely blocked in control and TSC1 knockdown with all other inhibitors. Furthermore, AKT phosphorylation was abolished by alpelisib (*p* = 0.01), not significantly altered by torin-1, and induced by sirolimus (*p* = 0.002). Of note, AKT phosphorylation was increased in TSC1 knockdown cells compared to control cells after torin-1 treatment (*p* = 0.006) (Fig. 4d, Additional file 1 Fig. 5-8).

Immunofluorescence staining of phosphorylated S6 (Fig. 4e) showed lower activation in TSC1 knockdown cells treated with inhibitors compared to solvent control, supporting the results from the Western blot analysis. Alpelisib, as a PI3K inhibitor, was less effective in reducing S6 activation compared to the mTOR inhibitors.

## Discussion

Intramuscular or subcutaneous lipomas are rarely reported in patients with tuberous sclerosis [3–7]. Genetic testing of the lipomatous tissue in patients with tuberous sclerosis was to date not performed and the underlying mechanisms of lipoma formation in tuberous sclerosis are not yet clarified.

Our patient presented with an angiolipoma in the right gluteal region. Based on the observed loss of heterozygosity with a 79% allele frequency for the likely pathogenic *TSC1* variant in the patient’s lipoma, we concluded that the TSC1 deficiency is likely causative for the lipoma. Correspondingly, we observed a decreased TSC1 protein expression and an increased pathway activation by enhanced phosphorylation of p70S6K in isolated cells from the patient’s lipoma compared to healthy donor cells.

To better understand the mechanisms of lipoma formation, we investigated whether a TSC1 knockdown has an impact on proliferation and differentiation of adipocyte progenitors.

A significant link between lipomas and mTOR pathway disorders is evident in patients with PHTS. Loss of PTEN leads to hyper activation of the mTOR signaling cascade, and enhanced adipocyte proliferation and differentiation [9, 15]. In patients with PHTS, the prevalence of lipomas is reported as 39%, underscoring the importance of this pathway in this pathophysiology [16].

Our data shows that the downregulation of TSC1 increases proliferation in adipocyte progenitors, providing further evidence for a role of TSC1 deficiency in lipoma development. Consistent with this, we observed increased phosphorylation of S6 in TSC1 knockdown cells, indicating mTOR pathway activation. Interestingly, we did not observe any pronounced effect on adipocyte differentiation in vitro. We propose that the primary mechanism underlying lipoma formation in tuberous sclerosis is enhanced proliferation resulting from TSC1 deficiency, while adipogenic differentiation may not be a contributing factor.

As previously described in the literature, some patients with diffuse lipomatosis associated with tuberous sclerosis have undergone surgical resection, however, a high rate of postoperative local recurrence has been reported, which was also observed in the case of the patient presented in our study [3–7]. Therefore, we sought to investigate in vitro whether a therapeutic approach using inhibitors could offer a potential treatment option for these patients. We previously observed that the PI3K inhibitor alpelisib was more potent in inhibiting PTEN haploinsufficient lipoma cell growth compared to sirolimus [9]. Furthermore, patients with adipose tissue overgrowth resulting from mosaic activating PI3K mutations have been effectively treated with alpelisib [17].

In TSC1 knockdown cells, treatment with the mTORC1 inhibitor sirolimus, the mTORC1 and mTORC2 inhibitor torin-1, and the PI3K inhibitor alpelisib led to reduced proliferation and viability/metabolic activity. While the effects on proliferation appeared more pronounced in TSC1 knockdown cells compared to controls without TSC1 knockdown, the effects on metabolic activity were similar for the inhibitors with an exception of sirolimus, which showed more specific inhibition of metabolic activity in TSC1 knockdown cells. Inhibition of the mTOR pathway was confirmed by decreased phosphorylation of downstream targets p70S6K and S6. Phosphorylation of AKT was reduced with torin-1 and alpelisib but induced with sirolimus. This induction is likely due to the alleviation of negative feedback via S6K1 upon mTORC1 inhibition, leading to increased AKT activation as has also been observed in PTEN-haploinsufficient lipoma cells [15]. The feedback usually occurs through the phosphorylation of IRS-1 by S6K1, leading to its degradation and thereby reducing the activation of AKT.

When mTORC1 is inhibited, the phosphorylation of rapamycin-insensitive companion of mTOR (RICTOR) by S6K1 decreases, leading to increased mTORC2 activity. Therefore, cell survival and proliferation could be promoted via increased phosphorylation of AKT through mTORC2 [18]. These mechanisms may lead to reduced efficacy of mTORC1 inhibitors like sirolimus in limiting tumor growth.

As mTORC2 typically creates a positive feedback loop by phosphorylating AKT [19], no increase would be expected for torin-1, which inhibits both mTOR complexes suggesting that dual inhibition of mTORC1 and mTORC2 might be more effective than targeting mTORC1 alone. However, torin-1 has not undergone evaluation in human clinical trials. The observation, that AKT (S473) phosphorylation was induced in TSC1 knockdown cells might be connected to a direct interaction of the TSC1/TSC2 complex with mTORC2 [20], but the mechanism remains elusive.

Our data indicate that rapamycin selectively reduces viability/metabolic activity of TSC1 KD cells while effects on control cells are less pronounced. In case of alpelisib and torin-1, both control and TSC1 KD cell viability decreased to the same extent.

Alpelisib, by inhibiting PI3K, may overcome resistance to mTORC1 inhibitors by directly targeting upstream signaling. In TSC1 knockdown cells, alpelisib reduced mTORC1 hyper activation, as shown by decreased proliferation and activation of downstream targets. While the more pronounced effects of torin-1, alpelisib, or combinations of alpelisib and sirolimus compared to sirolimus alone point to a more effective inhibition of tumor growth, this could also lead to side effects in vivo, especially given the less specific mode of action compared to sirolimus.

Clinical trials are increasingly exploring the use of dual PI3K/mTOR inhibitors in various cancer types. For instance, ongoing studies have evaluated the efficacy of NVP-BEZ235 in combination with other therapies for advanced solid tumors [21, 22].

Our observations suggest that mTOR pathway inhibitors may provide therapeutic benefits for patients with lipomas associated with tuberous sclerosis. mTORC1 inhibitors, such as sirolimus, are already an established treatment option for tuberous sclerosis patients and could potentially help reduce lipoma growth [1, 23]. For patients who do not respond to sirolimus alone, treatment with alpelisib, a combination of sirolimus and alpelisib, or a dual PI3K/mTOR inhibitor may offer additional benefits. However, there is currently no registered clinical trial of alpelisib in patients with tuberous sclerosis.

## Conclusions

The present study was designed to investigate the role of TSC1 deficiency in adipocyte progenitors to gain a deeper understanding of the mechanisms underlying lipoma development in patients with tuberous sclerosis. Our findings indicate that TSC1 downregulation promotes cell proliferation and activates mTOR signaling. In vitro treatment with mTOR inhibitors, such as sirolimus and torin-1, as well as the PI3K inhibitor alpelisib, reduced both proliferation rate and pathway activation. Our results suggest that mTOR pathway inhibitors may represent a potential therapeutic approach for patients with tuberous sclerosis-associated lipomas. Further research is necessary to determine whether treatment with pathway inhibitors can effectively reduce or even reverse lipoma growth in vivo.

## Supplementary Information

Below is the link to the electronic supplementary material.

Supplementary Material 1: Table S1: SVF cell cultures. Table S2: Primers used for qPCR. Table S3: Antibodies used for Western Blot (Wb) and immunofluorescence staining (IF). Fig. S1: Uncropped Western Blot (Wb) panels for Fig. 2c. Fig. S2: Uncropped Western Blot (Wb) panels for Fig. 3a, Wb 2-5. Fig. S3: Uncropped Western Blot (Wb) panels for Fig. 3a, Wb 6-8. Fig. S4: Uncropped Western Blot (Wb) panels for Fig. 3e Wb 9-13. Fig. S5: Uncropped Western Blot (Wb) panels for Fig. 3e Wb 14. Fig. S6: TSC1 did not affect adipocyte differentiation in SGBS cells. Fig. S7: Cell count is reduced after inhibitor Treatment in SGBS cells without TSC1 KD. Fig. S8: Uncropped Western Blot (Wb) panels for Fig. 4d (Replicate 1). Fig. S9: Uncropped Western Blot (Wb) panels for Fig. 4d (Replicate 2). Fig. S10: Uncropped Western Blot (Wb) panels for Fig. 4d (Replicate 3). Fig. S11: Densitometric analyses for Western Blots (Wb) 15-17 represented in Fig. 4d and Fig. S8-9.

Supplementary Material 2: Supplementary Table S4: Table containing data corresponding to the graphs presented in the manuscript.

## Data Availability

The datasets supporting the conclusions of this article are included within the article and its supplementary files. Further data is available from the corresponding author upon reasonable request.
