# Supplementary material for: In vitro study of TSC1 deficiency in preadipocytes: insights into development and treatment options for tuberous sclerosis related lipomatosis
Source: Orphanet J Rare Dis. 2026 Jun 12;21:216. doi: 10.1186/s13023-026-04442-y (PMC13262494; doi:10.1186/s13023-026-04442-y)
Supplement: Supplementary file 1 — Supplementary Material 1: Table S1: SVF cell cultures. Table S2: Primers used for qPCR. Table S3: Antibodies used for Western Blot (Wb) and immunofluorescence staining (IF). Fig. S1: Uncropped Western Blot (Wb) panels for Fig. 2c. Fig. S2: Uncropped Western Blot (Wb) panels for Fig. 3a, Wb 2-5. Fig. S3: Uncropped Western Blot (Wb) panels for Fig. 3a, Wb 6-8. Fig. S4: Uncropped Western Blot (Wb) panels for Fig. 3e Wb 9-13. Fig. S5: Uncropped Western Blot (Wb) panels for Fig. 3e Wb 14. Fig. S6: TSC1 did not affect adipocyte differentiation in SGBS cells. Fig. S7: Cell count is reduced after inhibitor Treatment in SGBS cells without TSC1 KD. Fig. S8: Uncropped Western Blot (Wb) panels for Fig. 4d (Replicate 1). Fig. S9: Uncropped Western Blot (Wb) panels for Fig. 4d (Replicate 2). Fig. S10: Uncropped Western Blot (Wb) panels for Fig. 4d (Replicate 3). Fig. S11: Densitometric analyses for Western Blots (Wb) 15-17 represented in Fig. 4d and Fig. S8-9. [file 13023_2026_4442_MOESM1_ESM.pdf]

SUPPLEMENTARY TABLES

Table S1: SVF cell cultures.

Name	Sex	Age at resection	Origin
SVF#1	Female	31	visceral
SVF#10	Female	29	visceral

Table S2: Primers used for qPCR.

target	FWD Primer	REV Primer	Probe
hTBP	TTG TAA ACT TGA CCT AAA GAC CAT TGC	TTC GTG GCT CTC TTA TCC TCA TG	AAC GCC GAA TAT AAT CCC AAG CGG TTT G
hHPRT	GGC AGT ATA ATC CAA AGA TGG TCA A	GTC TGG CTT ATA TCC AAC ACT TCG T	CAA GCT TGC TGG TGA AAA GGA CCC C
PPARy	GATCCAGTGGTTGCAGA TTACAA	GAGGGAGTTGGAAGG CTCTTC	TGACCTGAAACTTCAAGA GTACCAAAGTGCAA
Adipo-nectin	GGC CGT GAT GGC AGA GAT	CCT TCA GCC CCG GGT ACT	CGATGTCTCCCTTAGGAC CAATAAGACCTGG
FASN	GGCAAATTCGACCTTTCT CAGA	GGACCCCGTGGAATGT CA	CACCCGCTCGGCATGGCT ATCTT

Table S3: Antibodies used for Western Blot (Wb) and immunofluorescence staining (IF).

Primary antibody	Dilution	Distributor	Cat. no.
Hamartin/TSC1 (D43E2) Rabbit mAb #6935	1:1000 TBS-T 5%BSA (Wb)	CST	#6935
Phospho-p70 S6 Kinase (Thr389) (108D2) Rabbit mAb	1:1000 TBS-T 5%BSA (Wb)	CST	#9234
p70 S6 Kinase (49D7) Rabbit mAb	1:1000 TBS-T 5%BSA (Wb)	CST	#2708
Phospho-S6 Ribosomal protein (Ser235/236) (D57.2.2E) XP® Rabbit mAb	1:1000 TBS-T 5%BSA (Wb) 1:100 (IF)	CST	#4858
S6 Ribosomal Protein (5G10) Rabbit mAb #2217	1:1000 TBS-T 5%BSA (Wb)	CST	#2217
Phospho-AKT (S473) Rabbit mAb	1:1000 TBS-T 5%BSA (Wb)	CST	#4058
AKT antibody Rabbit polyclonal Ab	1:1000 TBS-T 5%BSA (Wb)	CST	#9272
PPAR gamma (81B8) Rabbit mAb	1:1000 TBS-T 5%BSA (Wb)	CST	#2443
alpha Tubulin (11H10) Rabbit mAb	1:2000 TBS-T 5%BSA (Wb)	CST	#2125
Fatty Acid Synthase Antibody	1:1000 TBS-T 5%BSA (Wb)	CST	#3189
Adiponectin (C45B10) Rabbit Monoclonal Antibody	1:1000 TBS-T 5%BSA (Wb)	CST	#2789
GAPDH (6C5) Mouse mAb	1:50,000 TBS-T 5% milk (Wb)	Merck	MAB374
Ki-67 (MIB-1) Mouse mAB	1:200 IF-buffer (IF)	Dako	M7240
**Secondary antibody**	**Dilution**	**Distributor**	**Cat. no.**
Polyclonal goat anti-rabbit immunoglobulin/HRP	1:2000 TBS-T 5% milk (Wb)	Dako	P0448
Polyclonal goat anti-mouse immunoglobulin/HRP	1:2000 TBS-T 5% milk (Wb)	Dako	P0447
Alexa Fluor 488 goat anti-mouse IgG H+L	1:1000 IF-buffer (IF)	Invitrogen	A11001

Fig. S1: Uncropped Western Blot (Wb) panels for Fig. 2c
Western Blots of SVF cells from healthy donors (SVF1, SVF10) and patient‘s SVF cells (P) in culture medium (CM) or serum free medium (0F).

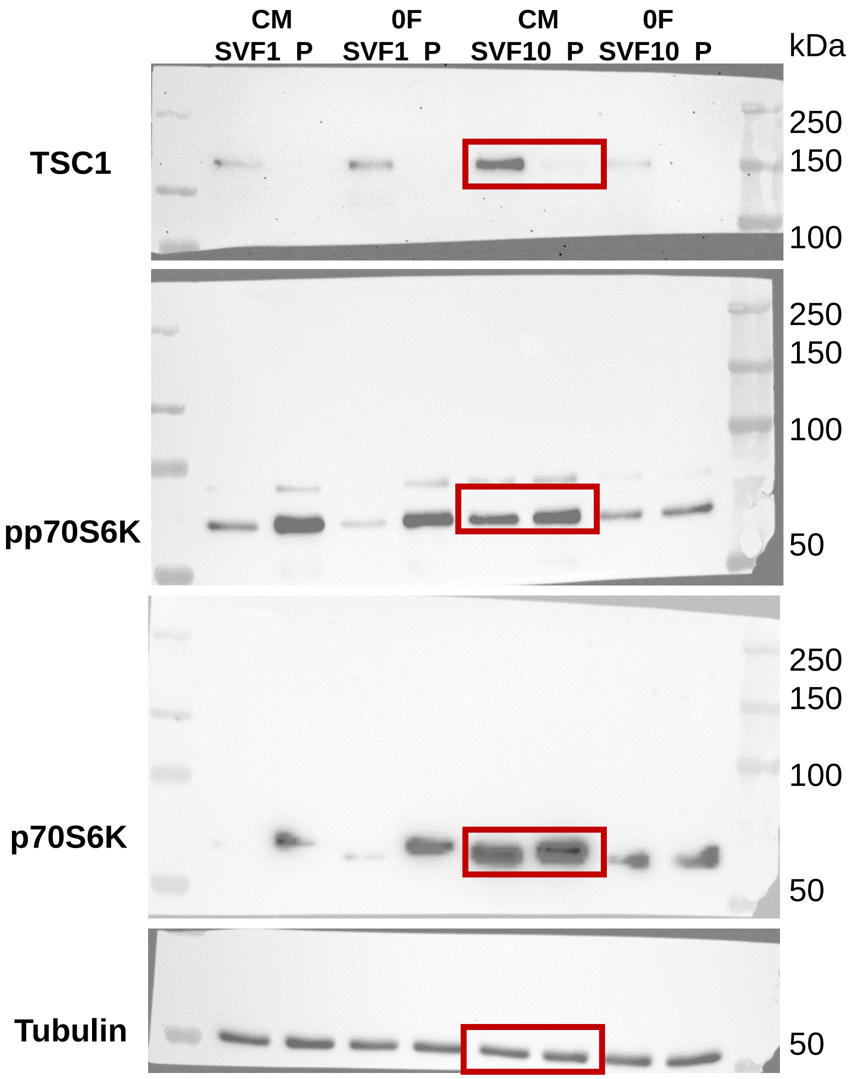

Wb 1:
Red boxes: represent in Fig. 2c

Fig. S2: Uncropped Western Blot (Wb) panels for Fig. 3a

TSC1 protein expression in control (c) and TSC1 knockdown (KD) SGBS cells.

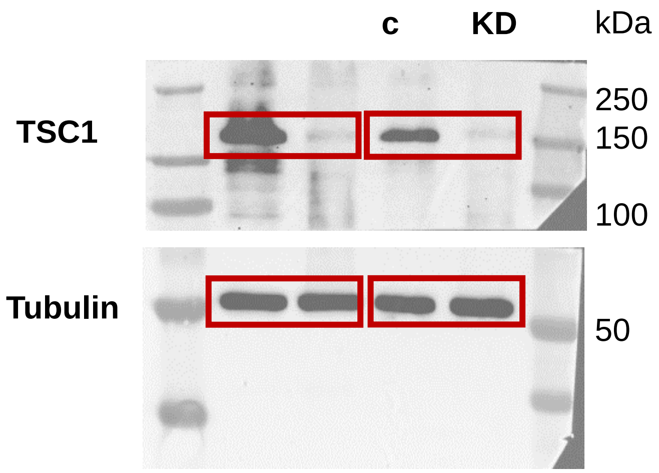

Wb 2:
Red boxes: represent in Fig. 3a (right) and used for densitometric analysis (both)

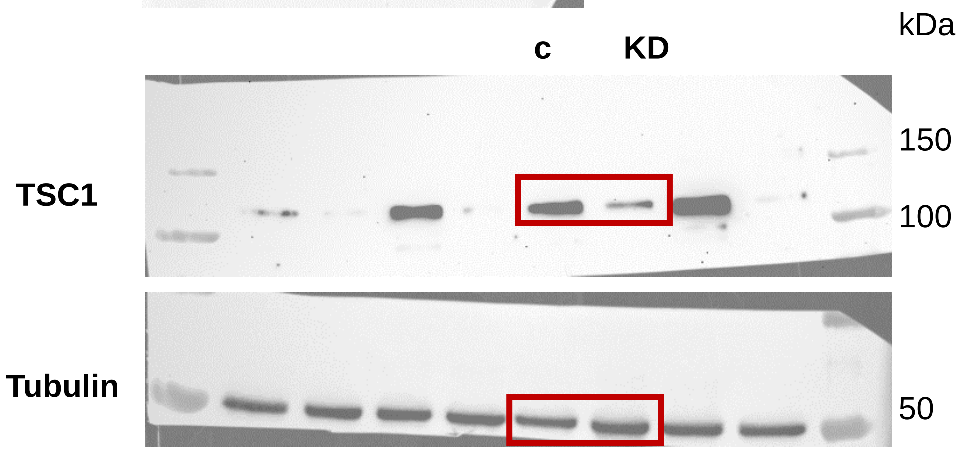

Wb 3:
Red boxes: used for densitometric analysis

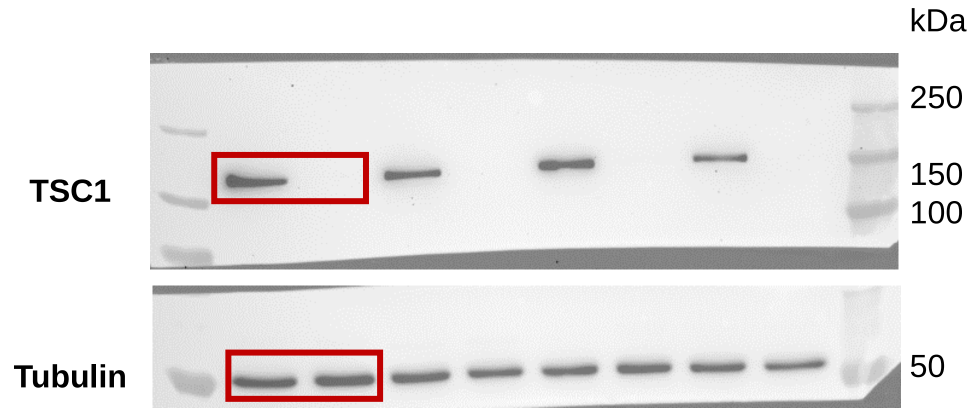

Wb 4:
Red boxes: used for densitometric analysis

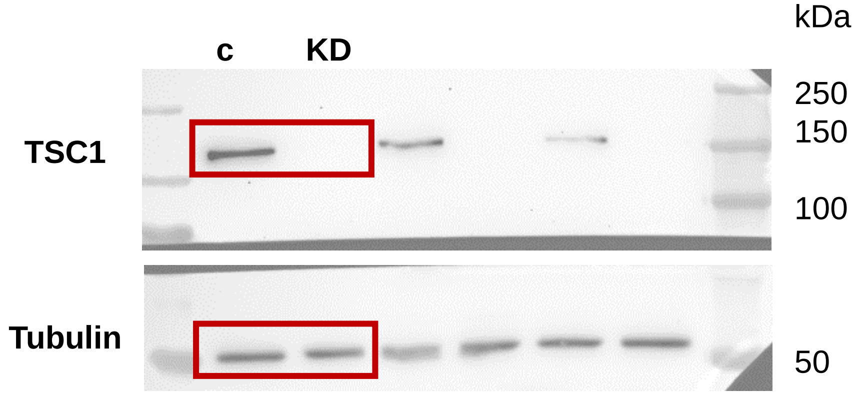

Wb 5:
Red boxes: used for densitometric analysis

Fig. S3: Uncropped Western Blot (Wb) panels for Fig. 3a
TSC1 protein expression in control (c) and TSC1 knockdown (KD) SGBS cells.

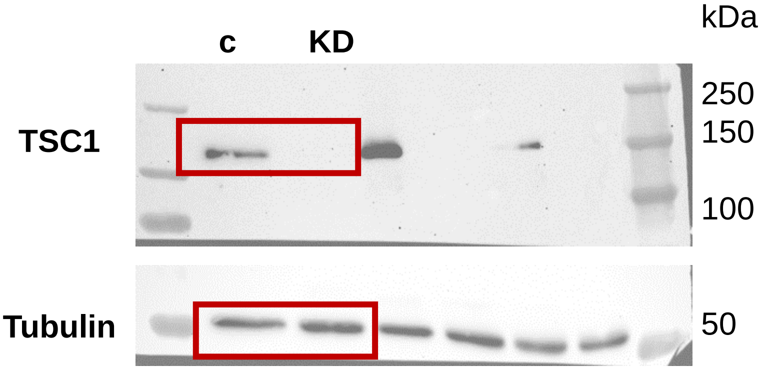

Wb 6:
Red boxes: used for densitometric analysis

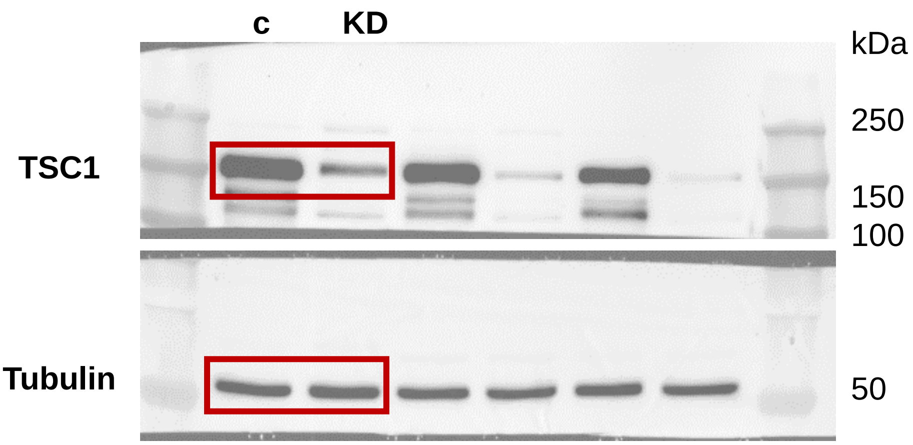

Wb 7:
Red boxes: used for densitometric analysis

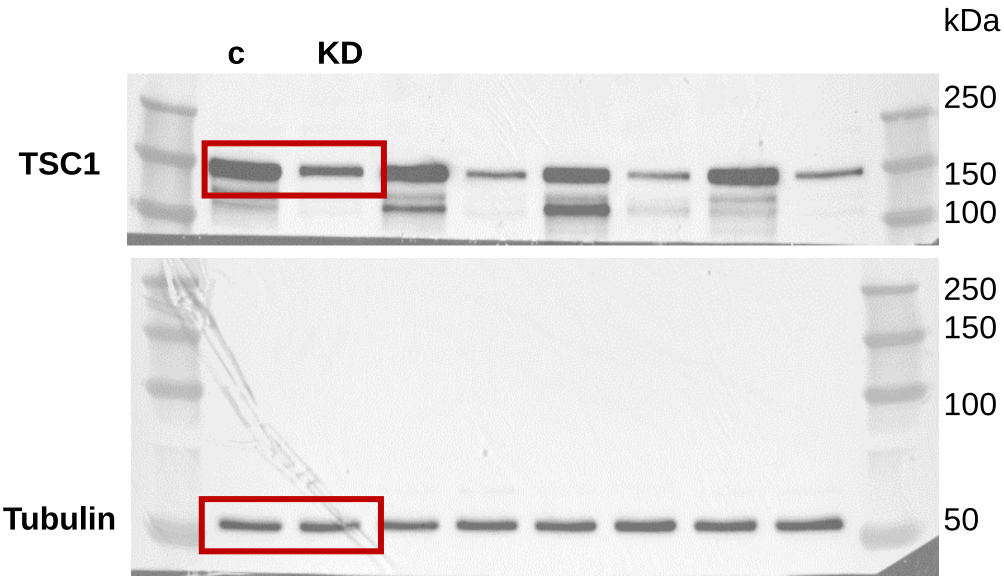

Wb 8:
Represent in Fig. 4d
Red boxes: used for densitometric analysis

Fig. S4: Uncropped Western Blot (Wb) panels for Fig. 3e
Phosphorylated (p) S6 protein expression in control (c) and TSC1 knockdown (KD) SGBS cells.

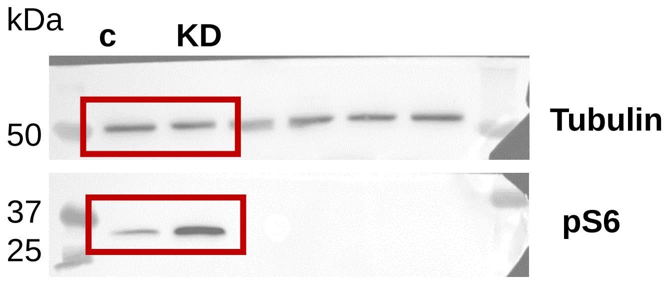

Wb 9:
Red boxes: represent in Fig. 3e and used for densitometric analysis
Tubulin is represent in Wb 5

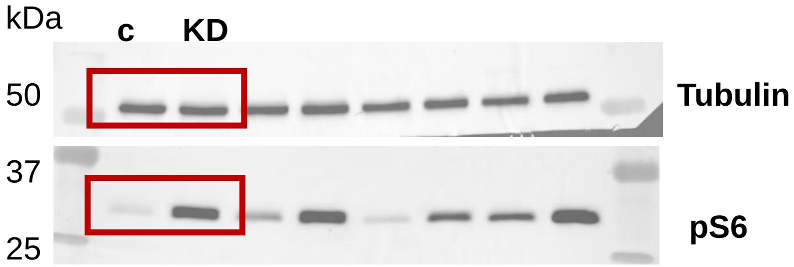

Wb 10:
Red boxes: used for densitometric analysis

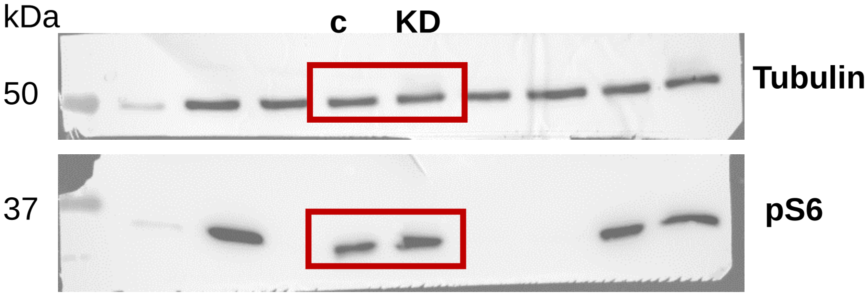

Wb 11:
Red boxes: used for densitometric analysis

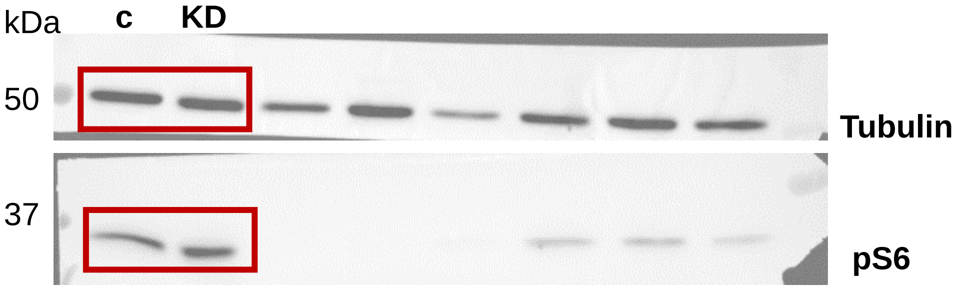

Wb 12:
Red boxes: used for densitometric analysis

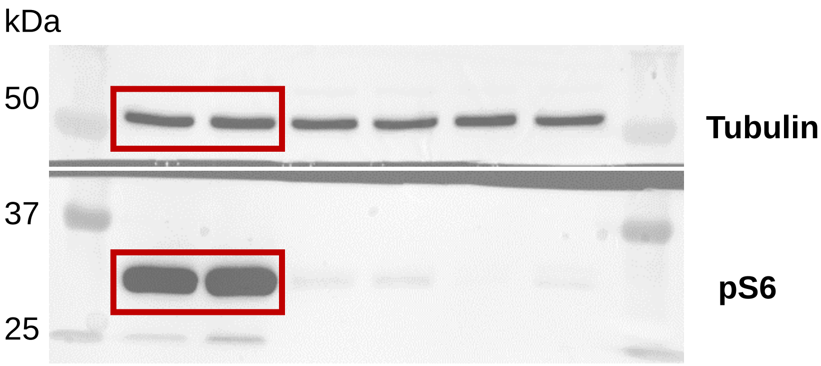

Wb 13:
Red boxes: used for densitometric analysis
Tubulin is represent in Wb 7

Fig. S5: Uncropped Western Blot (Wb) panels for Fig. 3e
Phosphorylated (p) S6 protein expression in control (c) and TSC1 knockdown (KD) SGBS cells.

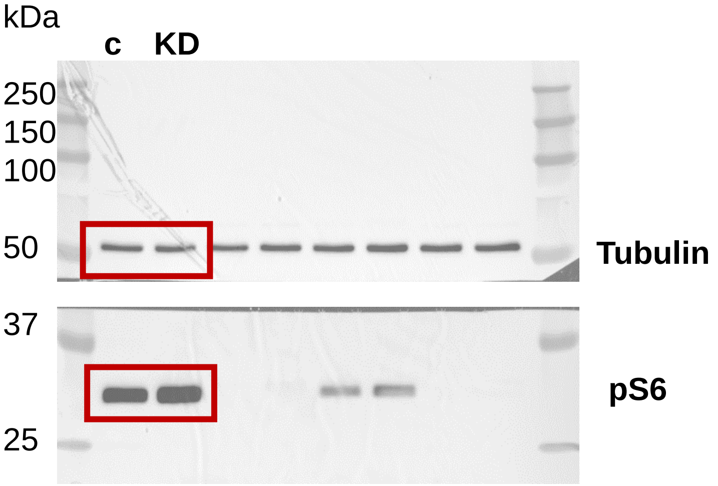

Wb 14:
Represent in Fig. 4d
Red boxes: used for densitometric analysis

Fig. S6

SUPPLEMENTARY FIGURES

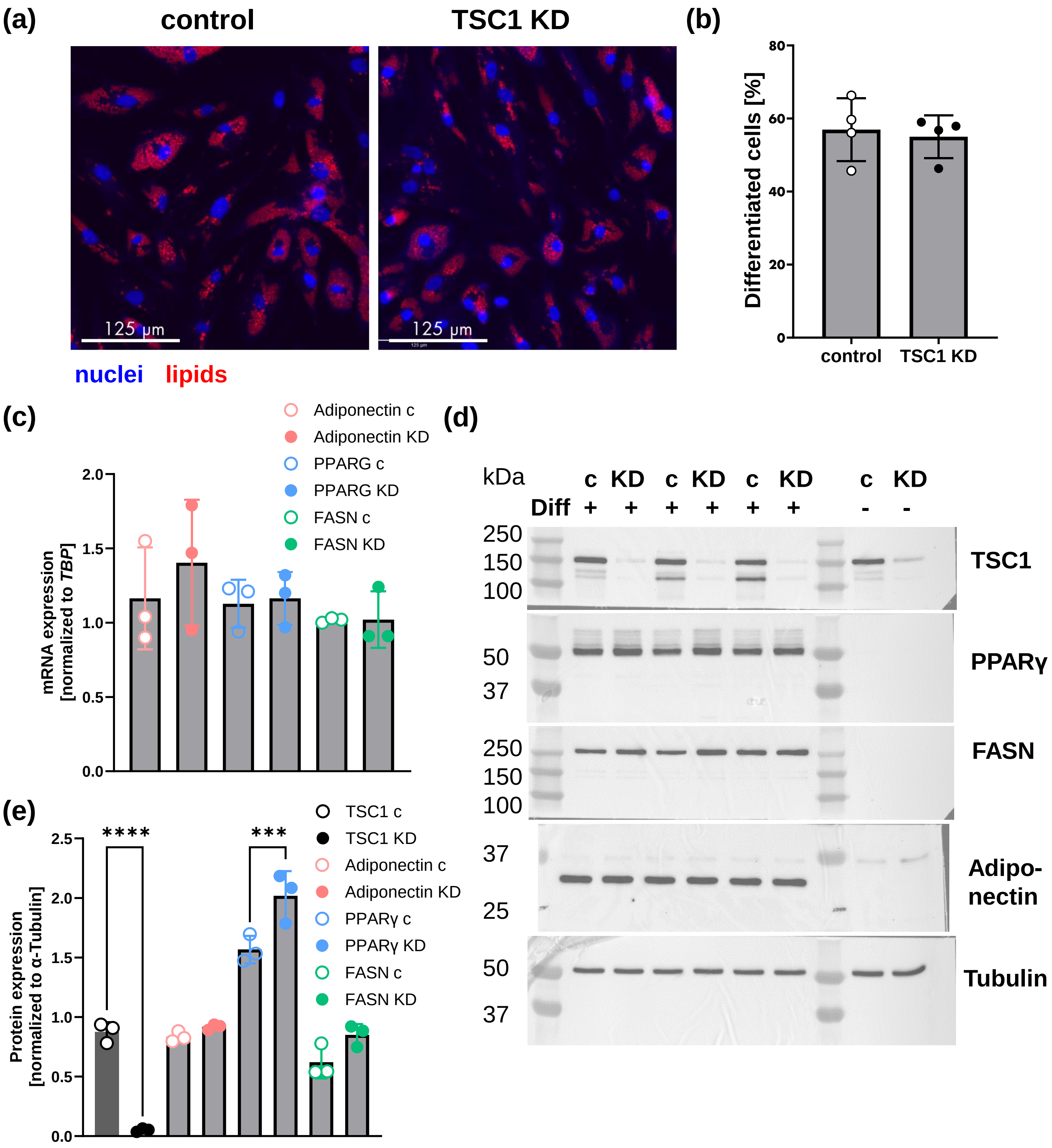

TSC1 did not affect adipocyte differentiation in SGBS cells.

(a) Microscopy images (200x magnification, blue: nuclei, red: lipids) and **(b)** quantification of Nile red lipid staining in differentiated control and TSC1 knockdown (KD) SGBS cells showed no difference in adipogenic potential (n=4, mean±SEM, p=0.737, determined via paired t-test). **(c)** We observed no difference in mRNA expression of adipocyte differentiation markers *adiponectin* (p=0.614), *PPARG* (p=0.997) and *FASN* (p>0.999) between differentiated control (c) and TSC1 knockdown (KD) cells (n=3, mean±SEM). **(d)** Western blot and **(e)** densitometric analyses of differentiated control (c) and TSC1 knockdown (KD) SGBS cells show persistent downregulation of TSC1 protein after 8 days of adipogenic induction (p<0.0001). Adiponectin was not (p=0.83), but PPARγ (p=0.0004) and FASN (p=0.07) protein were slightly increased in differentiated TSC1 KD cells (Diff +). Undifferentiated cells were shown as negative controls (Diff -). p-values for (c) and (e) were determined via one-way ANOVA followed by Šidáks multiple comparisons test.

Fig. S7

Control without TSC1 KD

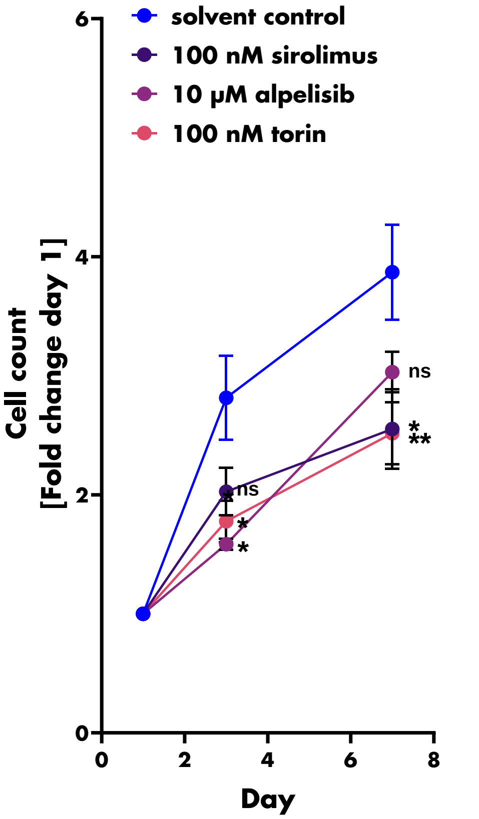

Cell count is reduced after inhibitor Treatment in SGBS cells without TSC1 KD. Hoechst nuclei staining of inhibitor treated SGBS cells: Cell count in treated cells was decreased after 3 and 7 days of inhibitor treatment compared to solvent control (n=7 for solvent control and sirolimus, n=3 for alpelisib, n=4 for torin-1, *p<0.05, **p<0.01, mean±SEM). p-values were determined via one-way ANOVA followed by Dunnett’s multiple comparisons test.

Fig. S8: Uncropped Western Blot (Wb) panels for Fig. 4d (Replicate 1)
Western Blots of control (c) and TSC1 knockdown (KD) SGBS cells in solvent control (sol), 100 nM sirolimus (sir), 10 µM alpelisib (alp), 100 nM torin-1 (tor), and 100 nM sirolimus + 10 µM alpelisib (S+A)

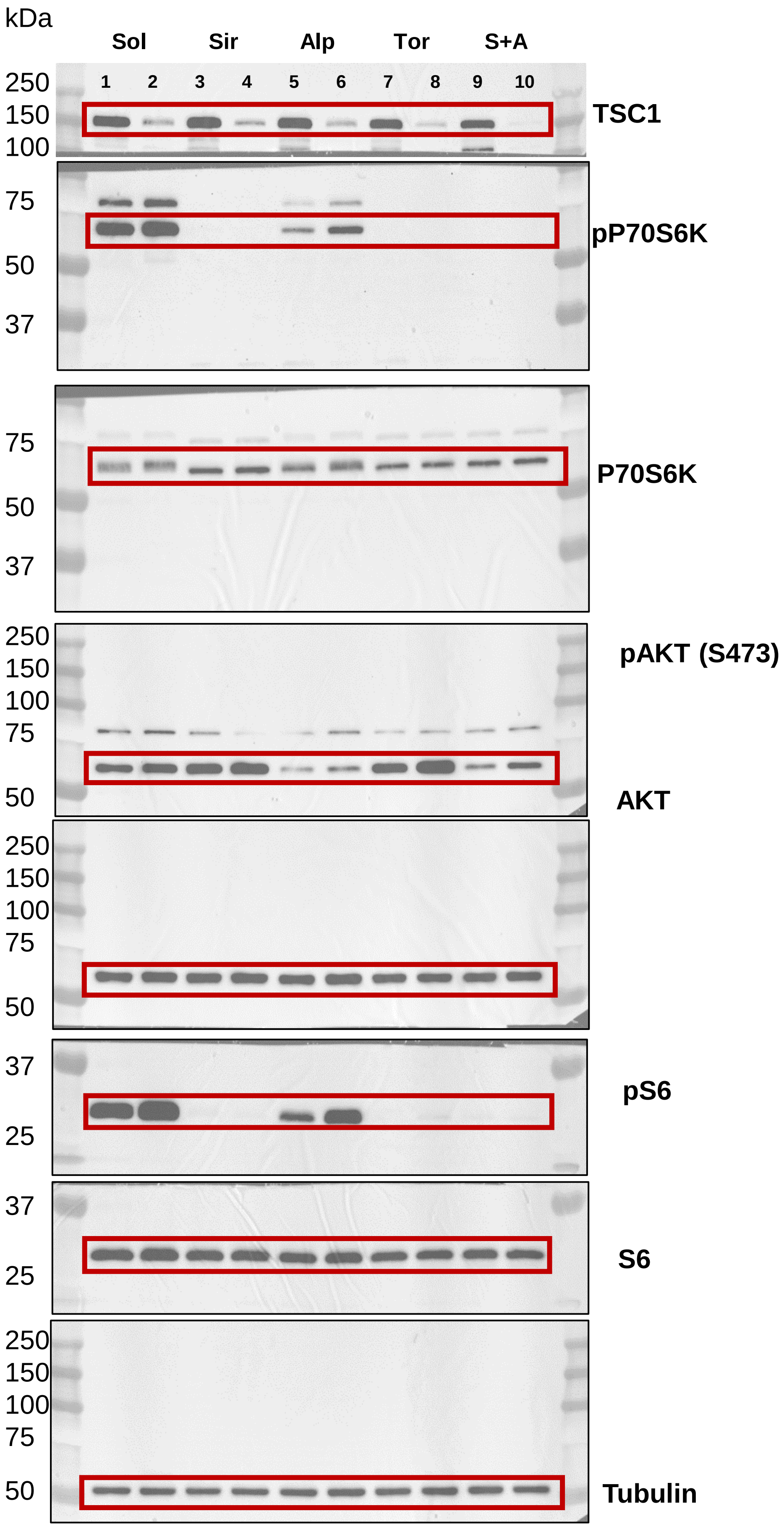

Wb 15:
Red boxes: Represent in Fig. 4d

1, 3, 5, 7, 9: c
2, 4, 6, 8, 10: KD

Fig. S9: Uncropped Western Blot (Wb) panels for Fig. 4d (Replicate 2)
Western Blots of control (c) and TSC1 knockdown (KD) SGBS cells in solvent control (sol), 100 nM sirolimus (sir), 10 µM alpelisib (alp), 100 nM torin-1 (tor), and 100 nM sirolimus + 10 µM alpelisib (S+A)

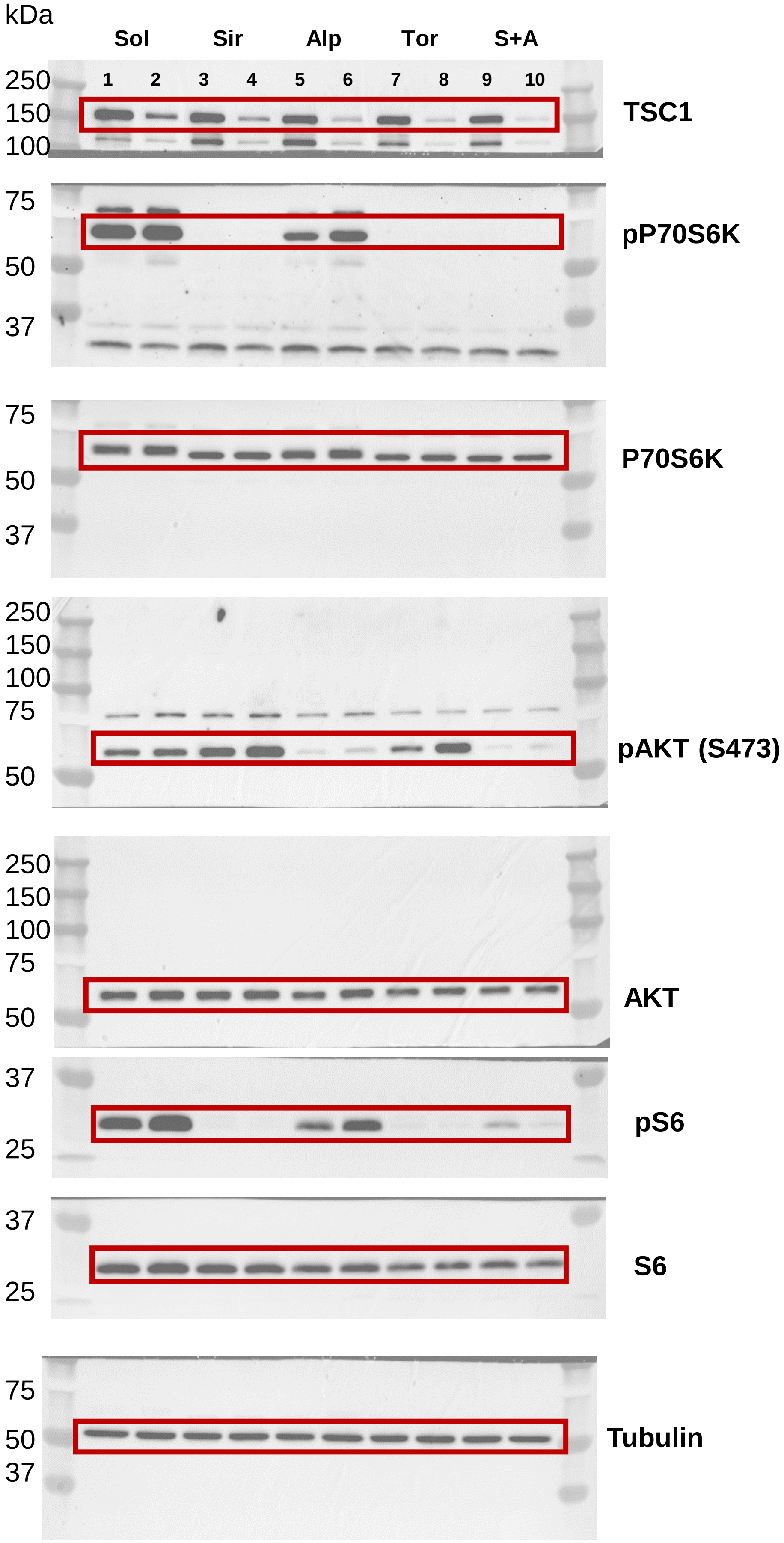

Wb 16:
Red boxes: used for densitometric analysis

1, 3, 5, 7, 9: c
2, 4, 6, 8, 10: KD

Fig. S10: Uncropped Western Blot (Wb) panels for Fig. 4d (Replicate 3)
Western Blots of control (c) and TSC1 knockdown (KD) SGBS cells in solvent control (sol), 100 nM sirolimus (sir), 10 µM alpelisib (alp), 100 nM torin-1 (tor), and 100 nM sirolimus + 10 µM alpelisib (S+A)

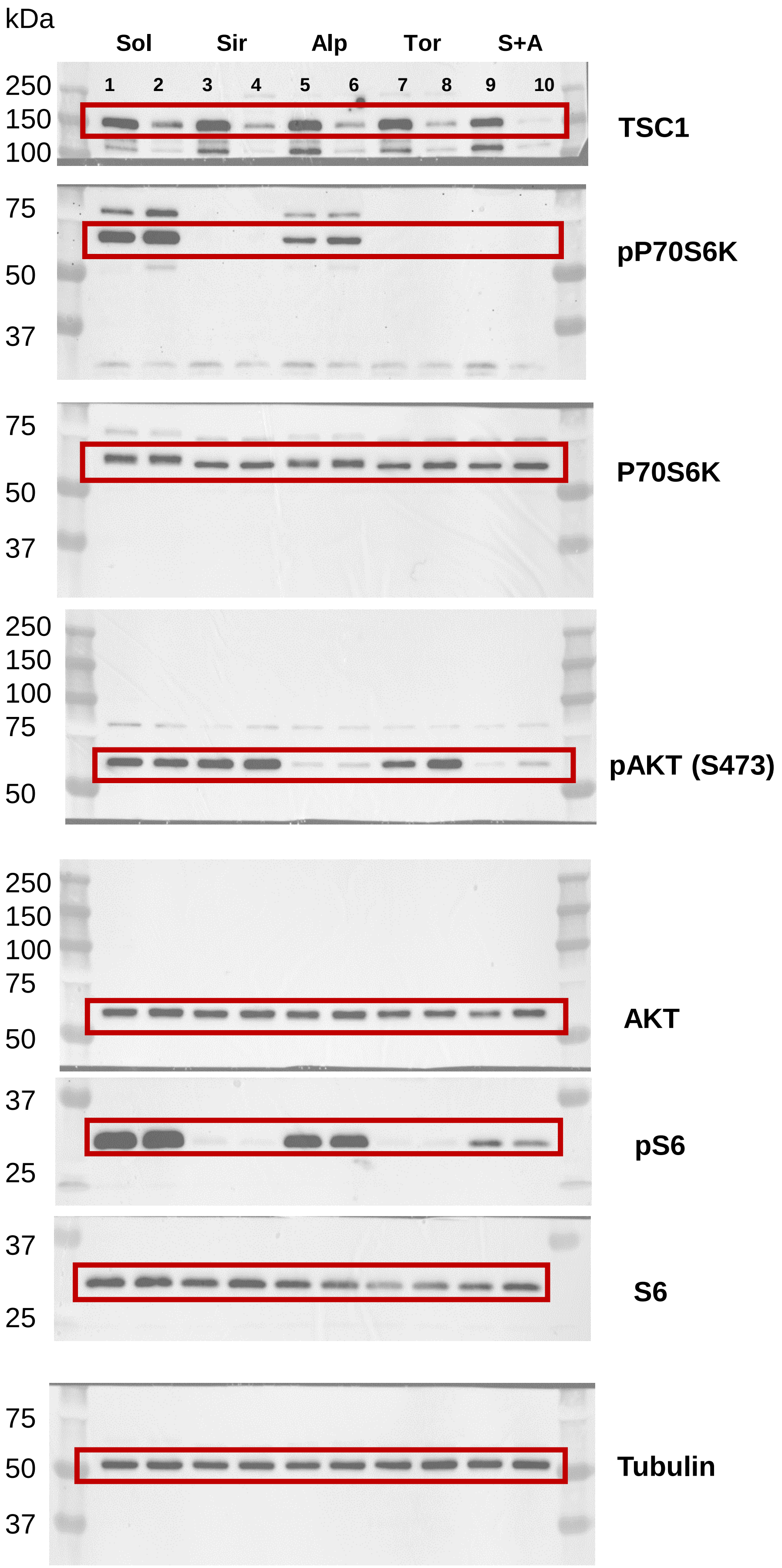

Wb 17:
Red boxes: used for densitometric analysis

1, 3, 5, 7, 9: c
2, 4, 6, 8, 10: KD

Fig. S11

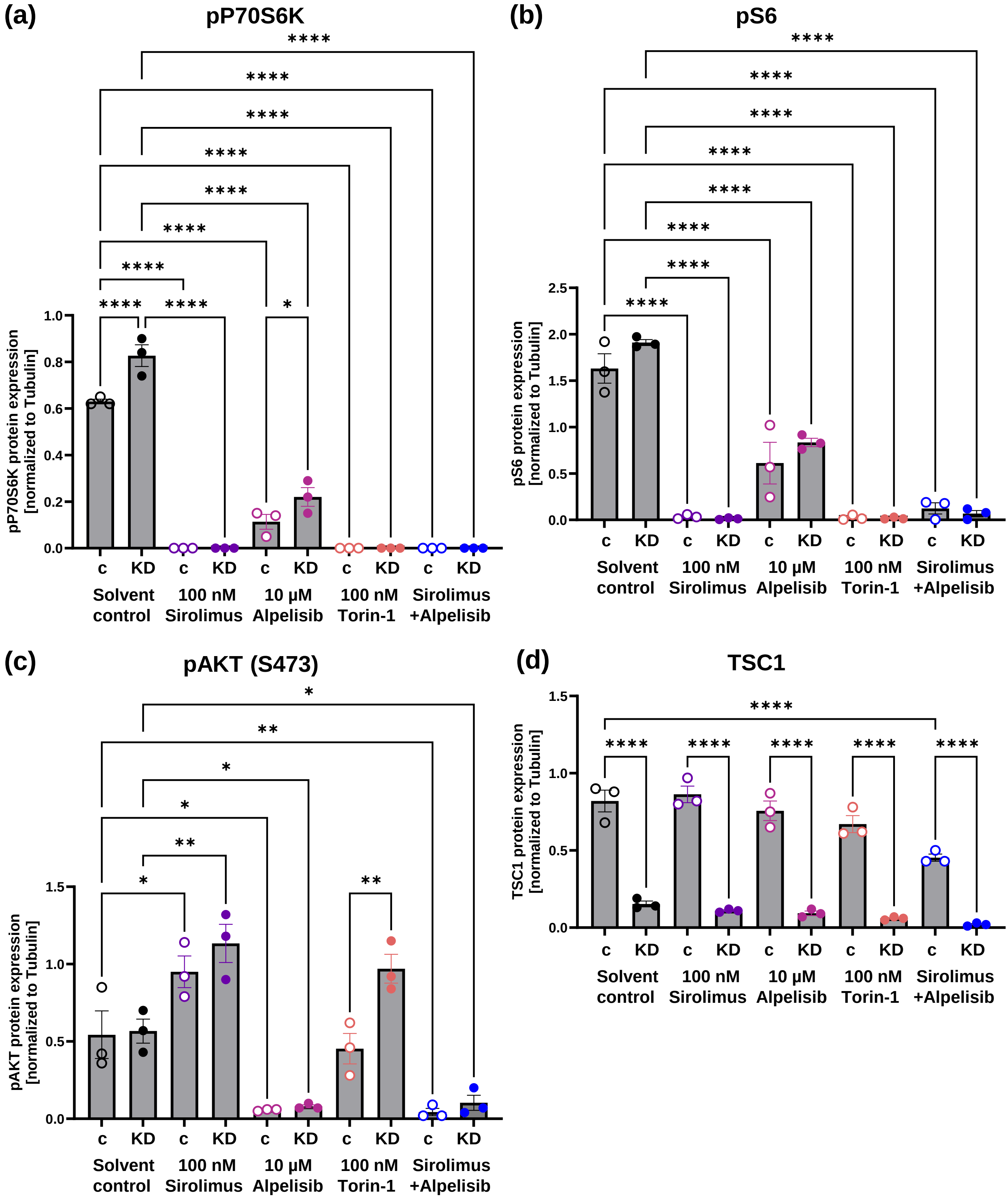

Densitometric analyses for Western Blots (Wb) 15-17 represented in Fig. 4d and Fig. S8-9
Analyses of Western blots of control (c) and TSC1 knockdown (KD) SGBS cells in solvent control, 100 nM sirolimus, 10 µM alpelisib, 100 nM torin-1, and 100 nM sirolimus + 10 µM alpelisib for **(a)** Phosphorylated P70S6K (pP70S6K), **(b)** Phosphorylated ribosomal protein S6 (pS6), **(c)** Phosphorylated AKT (pAKT), and **(d)** TSC1, (normalized to α-Tubulin, n=3, mean±SEM). p-values were determined via one-way ANOVA followed by Šidáks multiple comparisons test *p<0.05, **p<0.01, ****p<0.0001.
